# Cognitive outcome in children and adolescents treated for acute lymphoblastic leukaemia with chemotherapy only

**DOI:** 10.1111/j.1651-2227.2008.01055.x

**Published:** 2009-01

**Authors:** G Elisabeth Lofstad, Trude Reinfjell, Knut Hestad, Trond H Diseth

**Affiliations:** 1 Neuropsychological Clinic, Department of Psychology, Norwegian University of Science and TechnologyTrondheim, Norway; 2 Regional Centre for Child and Adolescent mental health, Department of Neuroscience, Norwegian University of Science and TechnologyTrondheim, Norway; 3 Neuropsychological Clinic, Department of Psychology, Norwegian University of Science and TechnologyTrondheim, Norway; 4 Section for Child and Adolescent Psychiatry, Paediatric Clinic, Rikshospitalet, University HospitalOslo, Norway

**Keywords:** Acute lymphoblastic leukaemia, Chemotherapy, Children, Cognitive functioning

## Abstract

Objective: To examine cognitive outcome in children and adolescents with acute lymphoblastic leukaemia (ALL) in remission, treated with central nervous system prophylactic chemotherapy only.

Method: Thirty-five children and adolescents, age 8.4–15.3 years in long-term remission from ALL, 4.2–12.4 years post diagnosis, without relapse and no prediagnosis history of neurodevelopmental disorder were compared with 35 healthy controls matched for gender and age, on measures of intellectual functioning Wechsler Intelligence Scale for Children-Third Edition (WISC-III).

Results: All but two of the ALL survivors treated by chemotherapy only obtained WISC-III Total Intelligence Quotient (IQ) scores in the normal range (M = 95.3), but their scores were significantly below levels for their matched controls and below normative standards for WISC-III. The difference between patients and controls was significant at the p < 0.001 level for the following measures: Total IQ, Verbal IQ, Verbal Comprehension Index, Freedom from Distraction Index and three verbal subtest scores.

Conclusion: The results indicate long-term sequelae in global cognitive functions, and indicate that verbal function, processing speed, attention and complex visual-spatial problem solving may be affected in the chemotherapy only group.

## INTRODUCTION

Acute Lymphoblastic Leukaemia (ALL) is the most common childhood malignancy, accounting for about 75% of all leukaemias and 25% of childhood cancers, with an incidence of 3.9/100.000 and a peak incidence at the age of 3–4 years (1). This is a disease of the lymphoid cells, where malignant white blood cells migrate via the circulatory system to virtually all organ systems, including the central nervous system, where the blood-brain barrier creates a sanctuary for cancer cells.

Current treatment commonly lasts for 24–30 months. All protocols include central nervous system prophylactic treatment to prevent central nervous system relapse. Treatment is based on complex multi-agent combinations of chemotherapies; the intensity of the therapy is determined according to risk groups, defined by graded risk for relapse and long-term sequelae. Some very high-risk cases also involve the use of cranial radiation therapy (CRT).

Treatment protocols are frequently changed in order to maximize long-term event-free survival and minimize long-term sequelae. Research on neurocognitive long-term outcome is closely interwoven with the ongoing treatment refinements. The introduction of central nervous system prophylactic CRT was followed by an increase in the five-year event-free survival rate from below 20% in 1960 to about 75–85% today (2). Unfortunately, CRT was documented as being significantly related to neurotoxicity with neurocognitive and neurobehavioral long-term sequelae, with younger children and particularly young girls particularly vulnerable (3,4). Through advances in central nervous system-directed chemotherapy, the use of CRT has been gradually replaced by intensified chemotherapy. In the Nordic countries, more than 90% are now treated with chemotherapy only (1). Recent chemotherapy only protocols often employ simultaneous administration of different groups of drugs, commonly including nucleoside analogs, glucocorticoids and antifolates, all of which are suspected of causing delayed neurotoxicity (5).

The cognitive functioning in long-term survivors of childhood ALL treated by chemotherapy only protocols has been evaluated by comparison to different groups, but results have been inconclusive. Earlier studies that focused on treatment effects often reported fewer and only subtle deficits in the chemotherapy only groups as compared to survivors treated with CRT (6,7), while other studies have reported no significant cognitive differences between such groups (8). Studies that have focused on both treatment and illness factors by including multiple samples (CRT treatment, chemotherapy only treatment, other illnesses such as non-central nervous system cancer, asthma and healthy controls) have shown a gradual effect on cognitive functions. ALL survivors treated with CRT showed the lowest cognitive levels, followed by chemotherapy only groups. Patients treated for other non-central nervous system cancers or asthma had higher scores than the chemotherapy only group, but scored below healthy controls (9,10). Gender and age at diagnosis have been documented to be significant moderators on cognitive functions (10), in studies by Brown et al. (11) only gender acted as a significant moderator. In a review by Moleski (12); all studies where cognitive function in survivor groups was compared to matched healthy controls reported significantly lower scores in survivor groups.

The lower scores on the WISC as documented in the literature might reflect impairment of both global and/or specific neurocognitive abilities. Studies that focused on outcomes for specific cognitive functions have reported significant impairment in Verbal IQ, Performance IQ, attention, information processing, executive functions, psychomotor skills, as well as verbal visual memory (13) and learning difficulties (14,15). Specific impairment in non-verbal function and freedom from distractibility has been documented (11), but specific impairment of Verbal IQ has also been documented (7).

The aim of this study was to explore cognitive outcomes in long-term survivors of childhood ALL treated with chemotherapy only in accordance with protocols developed in 1992 by the Nordic Society of Pediatric Hematology Oncology-ALL (NOPHO-1992), which is used in Denmark, Finland, Iceland, Sweden and Norway. The hypothesis is that survivors of childhood ALL treated with chemotherapy only protocols will have decreased level in cognitive functioning compared to healthy controls and test normative standards.

## MATERIALS AND METHODS

The present study consists of 35 children and adolescent long-term survivors of childhood ALL and 35 healthy controls matched for age, gender and socio-demographic variables (Table 1). The children and adolescents in the ALL survivor group were recruited from Rikshospitalet University Hospital in Oslo and St. Olav's University Hospital in Trondheim, Norway. The treatments were initiated from May 1992 through 1999 according to NOPHO-ALL 1992 protocols, with patients grouped as standard risk, intermediate risk, high-risk 1 group age below five years and high-risk 2 group age above five years. The very high-risk group was excluded. Patients were included if they met the following criteria: four years or more post-diagnosis, in continuous remission since the initial treatment with no relapses, completion of one single course of treatment without CRT. To maximize the homogeneity in the sample and minimize confounding effects of condition with a documented effect on cognition, patients in the very high-risk group treated with CRT or bone-marrow transplantation and patient with hospital record of central nervous system or B-cell leukaemia or other neurodevelopmental syndrome or diseases were not included. To prevent any culture or language bias in the test results, children from families from non-Nordic cultures and whose first language was not a Nordic language were not included. Fifty-one children and adolescents from the patient pools provided by the two hospitals met the inclusion criteria. Of 51 patients and their parents contacted by mail, 35 children (69%), 18 girls and 17 boys, agreed to participate in this study. This group had a mean age of 11.5 years (range 8.4–15.3 years) and was 4.2–12.4 years postdiagnosis. The 16 children who did not participate (13 girls and 3 boys) reported difficulty in finding time for the two-day testing period, whilst other families declined because they did not want to remind their children of their cancer treatment.

**Table 1:** Sociodemographic characteristics and treatment variables in 35 children treated for ALL and 35 healthy controls

	ALL	Healthy
Gender		
Girl: n (%)	18 (51.4)	18 (51.4)
Age at study in years		
Mean (SD)	11.5 (1.9)	11.6 (1.9)
Range	8.4–15.3	8.8–15.1
Family composition; n (%)		
Both parents	26 (74.3)	26 (74.3)
Parent with partner	3 (8.6)	4 (11.4)
Parent single	4 (11.4)	5 (14.3)
Unknown, not reported	2 (5.7)	0
Parent: age in year, mean (range)		
Mother	39.6 (30–54)	40.1 (30–52)
Father	43.1 (32–58)	43.0 (30–59)
Parent: education in years, mean (range)		
Mother	13.8 (10–19)	14.0 (9–19)
Father	14.3 (10–20)	13.7 (10–19)
Economy self evaluation		
Very good; n (%)	3 (8.6)	5 (14.3)
Good; n (%)	21 (60.0)	13 (37.1)
Average; n (%)	9 (25.7)	17 (48.6)
Poor	2 (5.7)	0
Home		
Own house, n (%)	33 (94.3)	34 (97.4)
Community		
Urban, n (%)	12 (34.3)	16 (45.7)
Rural, n (%)	23 (65.7)	19 (54.3)
Diagnosis and treatment		
Age at diagnosis in years		
Mean (SD)	3.8 (1.6)	
Median	3.6	
Range	1.6–7.5	
Time since diagnosis in years		
Mean (SD)	7.7 (1.9)	
Median	7.8	
Range	4.2–12.4	
Treatment protocols, n (%)		
Standard risk	14 (40.0)	
Intermediate risk	15 (42.9)	
High risk 1	4 (11.4)	
High risk 2	2 (5.7)	

No significantly differences were seen between the two groups.

The physically healthy children and adolescents in the comparison group were recruited from four public schools and matched by gender and age. The schools were selected to match demographics. As in the patient group only pupils from Nordic families with a Nordic first language without any known neurodevelopmental syndrome or disorders were recruited. The participation rate was 79%, with a mean age of 11.6 years and a range of 8.8 years to 15.1 years.

### Methods

Cognitive function was measured with the Wechsler Intelligence Scale for Children-Third Edition (WISC-III) Swedish version with Norwegian translation (16,17). All 13 subtests were administered: Information, Similarity, Vocabulary, Comprehension, Arithmetic, Digit Span, Picture Completion, Picture Arrangement, Block Design, Object Assembly, Coding, Symbol Search and Mazes. The Digit Span raw score was divided into a forward test of memory span and backward test of working memory. Age-adjusted scores for specific subtests were summarized and transformed into three intelligence scales; Total IQ, Verbal IQ and Performance IQ, and four composites index quotients; Verbal Comprehension Index, Perceptual Organization Index, Freedom from Distractibility Index and Processing Speed Index (Table 2).

**Table 2:** Independent sample *t*-test on cognitive outcome in 35 ALL survivors and 35 matched healthy control (HC) and one sample *t*-test for the ALL group compared to the Swedish WISC-III test norms

		ALL n = 35	HC n = 35	ALL vs. HC			ALL vs. norm data		
	Inc	Mean (SD)	Mean (SD)	*t*-score	p-value	Dif. 1	*t*-score	p-value	Dif. 2
IQ									
T IQ		95.3 (15.9)	109.4 (12.9)	−4.06	<0.001	14.1	−1.74	0.091	4.7
V IQ		94.1 (15.0)	110.1 (11.8)	−4.92	<0.001	15.9	−2.30	0.027	5.9
P IQ		97.9 (16.0)	106.4 (13.7)	−2.41	0.019	8.6	−0.79	0.434	2.1
Index									
VCI		94.7 (15.6)	108.6 (12.4)	−4.12	<0.001	13.9	−2.01	0.053	5.3
POI		99.4 (17.7)	106.7 (12.6)	−1.98	0.052	7.3	−0.19	0.850	0.6
FDI		94.4 (15.0)	108.8 (15.6)	−3.95	<0.001	14.4	−2.23	0.033	5.6
PSI		88.8 (14.9)	97.9 (15.6)	−2.49	0.015	9.09	−4.45	<0.001	11.2
Subtests									
Inf	tvc	10.1 (2.8)	11.0 (2.5)	−1.33	0.187	0.9	0.24	0.814	−0.1
Sim	tvc	8.7 (3.2)	11.6 (2.6)	−4.20	<.001	2.9	−2.51	0.017	1.3
Voc	tvc	8.1 (3.1)	10.4 (2.5)	−3.46	0.001	2.3	−3.65	0.001	1.9
Com	tvc	9.7 (3.3)	12.8 (2.5)	−4.53	<0.001	3.2	−0.62	.540	0.3
Ari	tvd	9.0 (2.5)	11.6 (3.0)	−3.97	<0.001	2.6	−2.38	0.023	1.0
DS	d	9.5 (3.2)	11.1 (3.5)	−1.94	0.057	1.5	−0.90	0.375	0.5
PC	tpo	9.9 (3.1)	9.6 (2.8)	−0.32	0.747	0.2	−0.27	0.789	0.1
PA	tpo	10.3 (3.2)	11.5 (2.9)	−1.58	0.119	1.1	0.59	0.559	−0.3
BD	tpo	9.6 (3.0)	11.7 (2.4)	−3.30	0.002	2.1	−0.78	0.437	0.4
OA	tpo	9.7 (3.9)	11.3 (2.1)	−2.45	0.028	1.7	−0.52	0.609	0.3
Cod	tps	9.1 (2.8)	10.4 (2.5)	−1.98	0.051	1.3	−1.89	.067	0.9
SS	s	7.1 (3.5)	8.9 (3.6)	−2.21	0.031	1.9	−4.93	<0.001	2.9
Maz		10.0 (4.2)	10.0 (3.8)	−0.03	0.976	0.0	−0.41	0.968	0.0
Raw									
DSf		8.03 (1.74)	8.92 (2.13)	−0.98	0.052	0.89			
DSb		5.19 (2.09)	5.70 (2.21)	−1.03	0.308	0.51			

Dif. 1: Group differences in mean scores for ALL survivors versus healthy controls (HC).

Dif. 2: Group differences in mean scores ALL compared to test normative means, ALL norm data; One sample *t*-test between ALL survivor group and the WISC-III normative mean.

Inc**:** This column shows the relation between subtests and the composite scores, each composite is marked by one letter: t: TIQ, Total IQ; v: VIQ, Verbal IQ; p: PIQ, Performance IQ; c: VCI, Verbal Comprehensions Index; o: POI, Perceptual Organization Index; d: FDI, Freedom from Distractibility Index; s: PSI, Processing Speed Index.

Subtests: Inf, Information; Sim, Similarity; Voc, Vocabulary; Com, Comprehension; Ari, Arithmetic; DS, Digit Span; PC, Picture Completion; PA, Picture Arrangement; BD, Block Design; OA, Object Assembly; Cod, Coding; SS, Symbol Search; Maz, Mazes; DSf, Digit Span forward and DSb, Digit Span backward.

Raw: in raw scores.

The parents were asked to fill in a standardized questionnaire regarding case history and demographic data based on a revised parental interview (18).

### Procedure

The study was approved by the Regional Ethics Committee of Medical Research. Written permission to contact the parents of children treated for ALL in their patient pools was provided by the leaders of the Paediatric Clinics at Rikshospitalet and St. Olav's Hospital. Written information about the project, parental and patient (adolescent) consent forms, and self-addressed, stamped envelopes were sent by ordinary mail to parents of children who met our inclusion criteria (n = 51). If forms were not returned within three weeks, parents were contacted by phone. All children for whom parents had provided informed consent were examined in a quiet room at the hospital where they had been treated for ALL.

To assemble the control group, the county borough councils for schools in one urban and one rural county were contacted to discuss the demographics of different schools, and to obtain permission to contact school headmasters in their respective counties. When the appropriate officials provided written informed consent, four school headmasters were contacted to provide informed consent, which was provided. Two Trondheim city headmasters (one from an elementary school, and one from a junior high school) were contacted first and asked to create a sample of two girls and two boys in each age group by drowing lots from gender-and age-specific (according to school year) pupil lists. The participants from these two schools and the children from the ALL group were divided by gender, ranged by age in months and matched by age. The age in months for ALL survivors without healthy matches was then recorded, after which headmasters from the rural county of Nord-Trøndelag were asked to select healthy matches by gender and age in months. Headmasters sent written information and consent forms to selected families, who were subsequently contacted by phone. Headmasters were instructed not to include pupils with known neurodevelopmental diseases diagnosed by the specialist health services. When informed consent was given by parents and adolescents, the WISC-III was administered in a quiet room at their respective schools. All tests were administered by the same individual, who is an experienced psychologist (T. R.).

## STATISTICS

The match between the two groups was evaluated by comparing demographical variables with independent sample *t*-tests or Pearson's chi-square. Cognitive outcomes for children and adolescents treated for ALL were compared to children and adolescents in the healthy control group by use of an independent sample t-test, and to mean scores from the WISC-III test norms using a one sample *t*-test. The interrelation between each of the global cognitive scores and the interrelation between the global cognitive scores and the subtest scores were explored by Pearson's correlation test. All results were evaluated using two-tailed analyses. The data were analysed using the Statistical Package for Social Sciences (SPSS, version 14.0, SPSS Inc., Chicago, IL, USA).

## RESULTS

### Group match

The demographical variables revealed no significant differences between patients and healthy controls (Table 1).

### Cognitive outcome

All but two of the ALL survivors obtained WISC-III Total IQ scores in the normal range (M = 95.3, Table 2). The ALL group scored significantly below the normative mean on the Verbal IQ, the Processing Speed– and Freedom from Distractibility-Indexes and the Symbol Search, Vocabulary, Similarity and Arithmetic subtests.

The between-group comparisons revealed significantly lower scores for the ALL group on six of the seven global measurements (Table 2). Verbal IQ, Total IQ, Verbal Comprehension Index and Freedom from Distractibility Index in the survivor group were 13 IQ points or more below healthy controls at the p < 0.001 level, while 7 of the 13 subtest scores were significantly below (p < 0.05) healthy controls. The group differences in the subtest scores were most striking with regards to Comprehension, Similarity and Arithmetic (p < 0.001).

There was a high correlation for the five most global composite scores; Total IQ, Verbal IQ, Performance IQ, Verbal Comprehension Index and Perceptual Organization Index in both groups (r = 0.63 to 0.98 in the survivor group, r = 0.50 to r = 0.97 in the control group). The Freedom from Distractibility Index was significantly correlated to these other scores in the ALL survivor group (p < 0.01). In the healthy group Freedom from Distractibility Index was significantly correlated to Total IQ, Verbal IQ and Performance IQ at the p < 0.05 level. This between group difference was only seen in one subtest the Digit Span, which was significantly correlated to global measures in the ALL survivor group but not in the healthy group. Arithmetic (the other subtest in Freedom from Distractibility Index) was significantly correlated to the five global measures in both groups. Processing Speed Index was not significantly correlated with the global scores in the ALL group, but it was significantly correlated to all five global measures in the healthy group (Total IQ, Verbal IQ, Performance IQ and Perceptual Organization Index at p < 0.01, and Verbal Comprehension Index at p < 0.05 level). This pattern in between group difference was found in both subtests (Coding and Symbol Search).

## DISCUSSION

The present study indicates that children and adolescents treated for ALL early in life by chemotherapy only show decreased global neurocognitive functioning. Their achievements on Total IQ, Verbal IQ, Verbal Comprehension Index and Freedom from Distractibility Index were all approximately 1 standard deviation below the matched controls. Group comparisons of the profiles from the WISC-III subtests scores indicate decreased level in verbal functioning, complex problem solving for arithmetic and visual spatial tasks, attention and processing speed. The majority of the reduced subtests are characterized by heavier reliance on higher mental or frontal lobe functions, which may represent a shared factor. Processing speed was also significantly lower in the ALL group, but their Processing Speed Index scores were not significantly related to their scores on the other global measures. The ALL survivors’ cognitive functions were also significantly below the test normative data for Verbal IQ, Processing Speed Index, Freedom from Distractibility Index and four subtests.

The results of the present study correspond to earlier documentation. In the review by Moleski (12) most studies documented cognitive outcome within normal range; but all studies enlisting sibling controls found decline in intellectual, neuropsychological, or academic achievement; the mean IQ value for healthy siblings of ALL patients was approximately 112–113. Previous studies including healthy controls are rare and the chemotherapy only patient groups have often been small, but the trend seems rather consistent. Among six studies reported over the last decade (7,9,13,15,19,20), four have reported a lower group mean Total IQ in ALL survivors of 8.3 to 22.2 IQ points compared to matched healthy controls (9,13,15,20). One study with only girls reported a lower group mean of 6.6 (19) in the survivor group, while another study reported 4.5 points lower Total IQ in the survivor group (7), although the healthy control group appeared to be something between a matched group and a larger test normative group. Five of these studies reported Total IQs from 97.6 to 107.2 among the ALL survivor groups (7,9,15,19,20), but all the control groups scored above Total IQ = 110. The largest group difference was reported in the study with the lowest Total IQ in the healthy group (Total IQ = 104) (13). In a study including 132 ALL survivors treated by chemotherapy only, Von Der Weid et al. (10) reported significant effects of gender and age at diagnosis among the survivor group. The whole group scored at the same level as patients treated for solid tumours (where the treatment did not include central nervous system prophylaxis treatment). The ALL survivors with the best cognitive prognoses (boys or age at diagnosis above six years) obtained a Total IQ close to 110, or nearly 10 IQ points above the ALL survivors with poorer cognitive prognoses.

In our study the group differences of specific cognitive functions was most striking and consistent for verbal function (Verbal IQ) and attention (Freedom from Distractibility Index). Specific decreases in verbal functions have been documented previously by Kingma et al (7). The ALL survivors in their study performed significantly below a large Dutch normative group on Verbal IQ, but no other scores showed significant differences. However, these researchers reported the cognitive functioning from two groups of ALL survivors treated by two different chemotherapy only protocols, and found this effect in only in one of the chemotherapy treatment groups. This finding of specific deficits in verbal function contrasts to findings from Brown (11), who reported specific deficits in non-verbal tasks and attention (Freedom from Distractibility Index). Data from the present study indicate deficits in complex visual spatial problem solving as measured by Block Design and Object Assembly, but not in the less abstract Picture Completion and Picture Assembly subtests, which load highly for detail recognition. The ALL survivors in this study did not differ significantly from controls for rote memory of learned facts, recognition and field-dependent visual tasks. Other researchers have documented mild but consistent impairment in recall of visual figures, verbal stories and memory span (13). Attention deficit has also been documented (11,21), and in research on CRT groups it has been postulated as a possible core deficit that results in later generalized cognitive and learning deficits (6). The lower Freedom from Distractibility Index scores in our ALL survivor group were largely caused by poorer scores on the Arithmetic subtest, which may be highly related to global cognition. Impaired processing speed has been documented by Mennes et al. (22). In our study, ALL patients had significantly lower Processing Speed Index scores than normative test data and that of controls, indicating impairment in processing speed, but Processing Speed Index was not significantly correlated to the other global score, which suggests it may be a specific deficit. Possible specific deficits in other functions may be confounded by global cognitive deficits.

Documentation of delayed brain changes, most commonly in reduction in the white matter volume, calcification, changes in glucose utilization and abnormalities in event-related potential (ERP) (5,21,23), strengthen our hypothesis of decreased levels in cognitive functioning. The young brain undergoes rapid development with a large increase in white matter volume, particularly in the frontal lobe, but also in the right hemisphere and the brain network. Immature white matter in areas undergoing rapid development is thought to be especially vulnerable. These areas are also known to be important in complex neurocognitive functioning. The association between documented neurological sequelae and neurocognitive impairment is still unclear. Some researchers reported no correlation (24), while other studies reported significant correlation (21,23).

The mean IQ in the control group in the present study might seem high compared to normative standards, but corresponds to previous documentation of cognitive level in matched control group of healthy children. The girl: boy ratio at 13: 3 among the survivors who refused to participate, is unlikely to have occurred by chance. The test leader was not blinded to the subject's group connection; the ALL group was tested in their respective hospitals, while the healthy children were tested at their respective schools and the number of participants was relatively low. Altogether these factors may limit our ability to document smaller impairments, and to make generalizations from our results.

Nevertheless, this study does have significant strengths, comprised of the close match to the healthy control group for gender, age and socio-economic variables, the homogeneity of the groups, the inclusion of 69% of the cohort treated for childhood ALL in the two hospitals following the inclusion criteria, and the fact that all participants were tested by the same test leader who was an experienced clinical psychologist.

## CONCLUSIONS

The present data strongly supports the hypothesis that early childhood ALL treated by chemotherapy only influences subsequent brain development and is followed by cognitive sequelae. Even though the cognitive outcome is in the normal range, it represents a substantial decrease of as much as one standard deviation compared to matched healthy controls. Our study revealed decreases in attention, verbal function, complex visual spatial problem solving and processing speed. The data do not allow us to draw conclusions on possible function-specific deficits. The WISC-III subtests are constructed to show both global intelligence and specific function; as a result, subtest scores may be confounded by global cognition. Strong correlations between the subtest scores and the composite scores in the present study may indicate that the survivors’ lower subtest scores may be influenced by a lower level of global cognitive function. Only Processing Speed was not significantly related to other scores. Further conclusions on the effect of function-specific deficits require additional function-specific neuropsychological tests. The growing number of reports of group mean Total IQ scores above 110 obtained by siblings and other healthy matched groups and patient groups with good cognitive prognosis highlights the importance of not merely relying on normative data for evaluating cognitive outcome.

The accumulated data showing cognitive sequelae after childhood ALL treated with chemotherapy only must be taken into account by schools and health care providers. Reduced IQ is a well-known risk factor for mental health problems, psychosocial dysfunction and school problems. Intervention programs must be constructed for long-term follow up to limit the secondary effects of lower IQ. ALL survivors should be taught strategies to compensate for their deficits.

In forthcoming publications we will focus further on outcomes in specific areas of neurocognitive functions evaluated by neuropsychological tests and the effect of risk factors for cognitive sequelae identified for treatment with CRT.
